# Computer-Aided Protein Directed Evolution: a Review of Web Servers, Databases and other Computational Tools for Protein Engineering

**DOI:** 10.5936/csbj.201209008

**Published:** 2012-10-22

**Authors:** Rajni Verma, Ulrich Schwaneberg, Danilo Roccatano

**Affiliations:** a School of Engineering and Science, Jacobs University Bremen, Campus Ring 1, 28759 Bremen, Germany; b Department of Biotechnology, RWTH Aachen University, Worringer Weg 1, 52074 Aachen, Germany

**Keywords:** directed evolution, rational design, semi-rational design, diversity generation, focused library, mutational effect

## Abstract

The combination of computational and directed evolution methods has proven a winning strategy for protein engineering. We refer to this approach as computer-aided protein directed evolution (CAPDE) and the review summarizes the recent developments in this rapidly growing field. We will restrict ourselves to overview the availability, usability and limitations of web servers, databases and other computational tools proposed in the last five years. The goal of this review is to provide concise information about currently available computational resources to assist the design of directed evolution based protein engineering experiment.

## Background

Protein engineering comprises a large number of techniques applied to evolve or design protein with desired function [1]. The primary objective in any protein engineering experiment is to identify specific sequence changes and alter the protein for desired functional properties [1, 2]. Generally, two main approaches are used to design the novel proteins or enzymes: rational design and directed evolution. The first approach employs the information of protein structure and focuses mutagenesis to modify protein scaffolds (e.g. the active site of the biocatalyst). For this approach, the knowledge of the target amino acid is necessary and can be provided by visual inspection or *in-silico* prescreening [3]. Both cases depend on the nature of the problem and show high success rate only for the prediction of single or double site mutations. Indeed, multiple mutations involve cooperative effects on protein structure and function that are difficult to predict using the current computational screening methods as well.

A more challenging *de novo* design or redesign of synthetic protein or peptide uses solely structural information and folding rules of the proteins [4, 5]. Although the method offers broadest possibility to design novel fold and function, the success for large proteins is limited [6, 7]. The reasons rely on the limited number of three-dimensional protein structures (in particular membrane proteins) and the lack of unifying theory for protein folding mechanisms. Computational approaches based on microseconds to milliseconds atomistic [8–10] molecular dynamics (MD) simulations of protein folding have recently given some encouraging achievement for *ab-initio* folding of peptides and small proteins. In addition, the combined approach of quantum mechanics and molecular dynamics methods have shown the superior capability of physical based method to design new enzymatic reaction [11]. However, the application of these methods is still limited since they are considerably computational time demanding [12]. In this review, the approaches based on *de novo* design, quantum mechanics and molecular dynamics will not be covered. The reader can refer to different recent papers and reviews on these topics [13–16].

The second approach is the so-called directed evolution. The method is one of the most powerful approaches to improve or create new protein function by redesigning the protein structure [17]. It can, for example, improve activity or stability of biocatalyst under unnatural conditions (e.g. the presence of organic solvent) by accumulating multiple mutations [17, 18]. Directed evolution involves multiple rounds of random mutagenesis or gene shuffling followed by screening of the mutant library [19]. The preliminary knowledge of protein structure is not required in directed protein evolution. However, the structural information can focus and restrict the approach to specific subsets of amino acids (e.g. active site residues). A common problem of directed evolution methods is the limited distribution of generated sequence diversity that reduces the efficient sampling of functional sequence space [19, 20].

In summary, rational design via site directed or saturation mutagenesis and directed evolution via random mutagenesis are used as key tools in protein engineering. In both approaches, the sequence diversity is directly generated as point mutation, insertion or deletion within a single parental gene. Consequently, the improvement in the quality of rationally designed libraries and techniques for sequence space exploration and diversity generation is critical for future advances.

The combination of experimental and computational methods holds particular promise to tailor the proteins for tasks not yet exploited by natural selection [21, 22]. In fact, most of the computational tools or web servers for directed evolution utilize, when it is possible, structural data to assist library generation processes. Since it is impossible to test more than a very small fraction of vast number of possible protein sequences, it urges to have a directed evolution strategy for generating sequence libraries with the highest chance to have variants with desired enzymatic properties. Such libraries can be designed by applying the current knowledge of the protein response towards mutations and sequence-structure-function relationships.

Thermo stability, solvent effects (pH, ionic strength and co-solvents stability or tolerance) and enzymatic activity (as improvement in both binding affinity and catalytic activity) are the properties commonly targeted by protein engineering experiments. The first two properties are subtle to predict cause they usually involve amino acids distributed on the whole protein structure. For the enzymatic activity, different mutagenesis studies indicate that mutations, affecting certain enzyme properties (as substrate specificity, enantioselectivity and new catalytic activities) are mostly located into or near the active site [21]. Rational design approach is successful in targeting relevant active site residues for site-directed mutagenesis but less effective for important residues located in the second coordination sphere of the active site. For these cases, the combination of random mutagenesis and computer-aided protein directed evolution (CAPDE) approaches can provide a winning strategy. The application of computational methods in conjunction with directed evolution offers the exciting promise to generate libraries having high frequency of active and improved variants [23].

**Figure 1:**
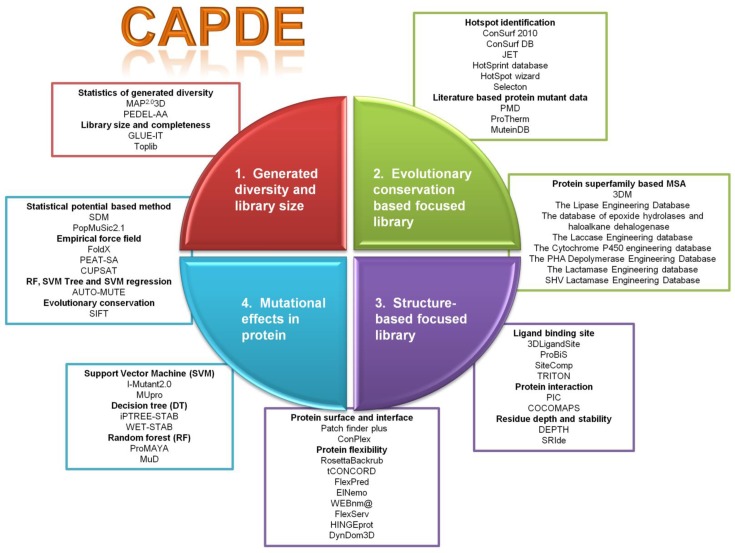
Schematic representation of four CAPDE approaches (as the quarters of the circle): (1) generated diversity and library size (in red), (2) evolutionary conservation based focused library (in green), (3) structure-based focused library (in purple) and (4) mutational effects in protein (in cyan). The servers, tools and databases associated with the approaches are shown in boxes.

In this review, for sake of clarity, the CAPDE approaches have been divided in four major areas, schematically represented in Figure 1. The first one comprises tools used for characterizing the library generated by mutagenesis methods mainly through the statistical approaches. The second and third areas are represented by tools that consider the evolutionary and structural information of the target protein to design the focused library. Multiple sequence or structure alignment (MSA) is the key approach used by these tools to identify variable or conserved positions in the target protein. The fourth part is dedicated to the tools for the prediction of mutational effects on protein structure and function. These tools and/or web servers are based on machine learning, statistical or empirical approaches and predict mutational effect on protein stability and/or activity by estimating the relative free energy changes [24].

This review is divided in four parts following the division of CAPDE approaches. It aims to provide the concise information about currently available CAPDE methods to assist and design directed evolution experiments with the final goal to enhance the probability of identifying mutants with desired properties. In particular, the reader will find a short overview and classification to the novel database, web server and other computational tools that can provide relevant information for the interpretation of experimental results and have been developed in the last few years in the field of molecular modeling of protein structure. Finally and as previously mentioned, we are not going to take in consideration the methods that involve physical approach based on QM/MM or MD simulations.

## Generated diversity and library size

The unbiased diversity generation followed by the screening of a statistically meaningful fraction of generated sequence space are fundamental challenges in directed evolution experiments [25]. The directed evolution strategy comprises two key steps: 1) generate diverse mutant libraries and 2) screen to identify the improved protein variants. The success of a directed evolution methods depends upon the quality of the mutant library. The challenges and advances to generate the functionally diverse libraries have been reviewed in past year [20, 26]. Computational tools can assist directed evolution in these two steps by *in-silico* analysis and screening of expected protein sequence space sampled by generated libraries (summarized in Table 1). Publicly available web servers, *MAP* (Mutagenesis Assistant Program) [25, 27] and *PEDAL-AA* [28] were developed to estimate the diversity at protein level in the library generated by random mutagenesis method.

**Table 1:** Summarizing computational tools to analyze amino acid diversity, size and completeness of the library generated by mutagenesis methods.

Approach	Name	Input	Case study examples	URL
Statistics of generated diversity	*MAP*^*2.0*^*3D* [25, 27]	Nucleotide sequence or protein structure.	Cytochrome P450BM-3, [25] D-amino acid oxidase, Phytase [27]	http://map.jacobs-university.de/submission.html
	*PEDEL-AA* [28]	Nucleotide sequence, mutation rate, library size, indel rate, nucleotide mutation matrix.	α-synuclein, Phosphoribosylpyrophosphate amidotransferase [33]	http://guinevere.otago.ac.nz/cgi-bin/aef/pedel-AA.pl
Library size and completeness	*GLUE-IT* [28]	Library size and randomization techniques.	Randomization scheme: NNK, NDT, NNB, NAY [28]	http://guinevere.otago.ac.nz/cgi-bin/aef/glue-IT.pl
	*TopLib* [32]	Probability required by library size and randomization techniques.	Randomization scheme: NNN, NNB, NNK, MAX [32]	http://stat.haifa.ac.il/∼yuval/toplib/

**Figure 2:**
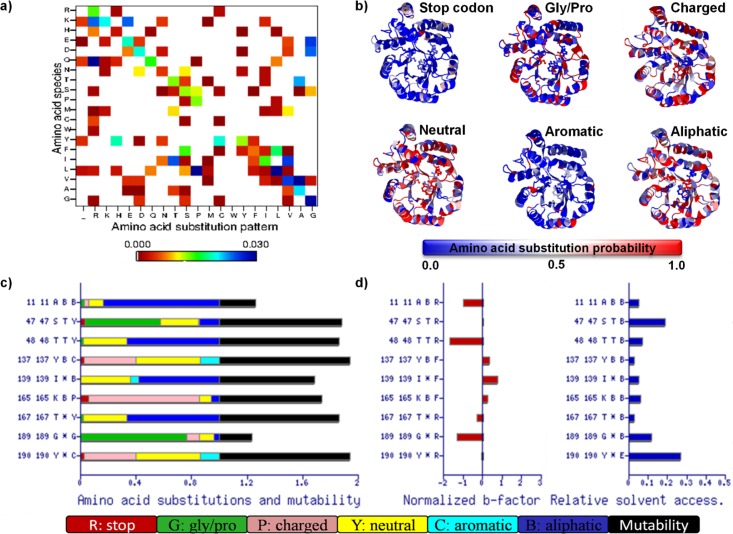
a) The *MAP*^*2.0*^*3D* analysis for the amino acid diversity generated by balanced epPCR (Taq (MnCl_2_, G=A=C=T) method. Y-axis shows the original amino acid species and the X-axis shows the amino acid substitution patterns. The *MAP*^*2.0*^*3D* analysis is restricted to the active site residues (Ala11, Ser47, Thr48, Tyr137, Ile139, Lys165, Thr167, Gly189, Tyr190). For this analysis, the amino acids are grouped into four classes according to their chemical nature (charged, neutral, aromatic and aliphatic) with stop codon ((structure disrupting) and glycine/proline (helix destabilizing) as separate classes. The probabilities of amino acid substitutions were mapped on the protein sequence and structure (PDB Id: 1NAL) of *N*-acetylneuraminic acid and represented in b and c, respectively. b) The Jmol [31] applet is used for the visualization of amino acid substitution patterns using RWB (Red-white-blue) color gradient scheme and active site residues as sticks. Y-axis shows sequence id, PDB id, amino acid name and in c) secondary structure elements (T: hydrogen bonded turn and bend, *: loop or irregular structure), d) normalized Cα b-factor to differentiate flexible (F) and rigid (R) residues, and e) relative solvent associability to identify exposed (E) or buried (B) residues.

*MAP* [25] takes nucleotide sequence as input and assists to design better directed evolution strategy by providing the statistical analysis of random mutagenesis methods on protein level. The capabilities of *MAP* was recently extended in *MAP*^*2.0*^*3D* [27] server that predicts the residue mutability resulted by the mutational bias of random mutagenesis methods and correlates the generated amino acid substitution patterns with the structural information of the target protein. In this way, the server offers the possibility to analyze at sequence and structural level the effects of the limited mutational preferences of random mutagenesis methods [25]. The capability of the server was illustrated by the *in-silico* screening of different enzymes and the predicted results were in agreement with the experimental results [27, 29, 30]. Figure 2 shows an example of the *MAP*^*2.0*^*3D* output for active site residues of *N*-acetylneuraminic acid using epPCR method [27].

*PEDAL-AA* returns statistics, at amino acid level and for libraries generated by epPCR method, after providing the nucleotide sequence with library size, mutation rate, indel rate and nucleotide mutation matrix [28]. *CodonCalculator* and *AA-Calculator* are two algorithms developed by Patrik *et al*. to select an appropriate randomization scheme for library construction [28]. Two servers *GLUE-IT* and *GLUE* estimate amino acid diversity and completeness in the generated library. Finally, the *TopLib* [32] web server assists to design saturation mutagenesis experiment by predicting the size or completeness of the generated library with the user-defined codon randomization scheme using probabilistic approach.

## Evolutionary conservation based focused library

Multiple sequence or structure alignment (MSA) is the most common approach to identify functionally significant or evolutionary variable regions in protein [34]. In CAPDE, several servers and databases use MSA with the physical and structural information of protein or protein superfamilies. Table 2 contains a list of the tools considered in this review. *ConSurf 2010* [35] server provides the evolutionary conservation profiles of protein or nucleic acid sequence or structure by first identifying the conserved positions using MSA and then calculating the evolutionary conservation rate using an empirical Bayesian inference. *ConSurf-DB* [36] database make available the evolutionary conservation profiles of the available protein structures pre-calculated by *ConSurf* web server. The *3DM* [37] server performs structure based multiple sequence alignments (MSA) of the members of a protein superfamily and provides the consensus data combined with other useful information, like interactions and solvent accessibility, about amino acid positions in protein with published mutation data.

**Table 2:** Summarizing computational tools for evolutionary conservation based focused library generation.

Approach	Name	Description	Case study examples	URL
Hotspot identification	*ConSurf 2010* [35]	The web server performs MSA and calculates evolutionary conservation rate to identify conserved positions in protein or nucleotide sequence/structure.	GAL4 transcription factor [35]	http://consurf.tau.ac.il/
	*ConSurf DB* [36]	The database provides the predicted results of *ConSurf* [35] server for known protein structures.	Cytochrome c [36]	http://consurfdb.tau.ac.il/index.php
	*JET* [38]	The Evolutionary trace based method performs MSA on a set of homologous sequences (from PSI-BLAST) after Gibbs like sampling. The aligned homologous sequences are used to construct distance tree based on Neighbor Joining algorithm. The clustering method is parameterized to identify protein interface or core residues by taking into account the physical-chemical properties and evolutionary conservation.	DNA polymerase I, DNA transferase, allophycocyanin, Leucine dehydrogenase, β-trypsin proteinase, phosphotransferase, human CDC42 gene regulation protein, oncogene protein, signal transduction protein etc [38]	http://www.ihes.fr/∼carbone/data6/legenda.htm
	*HotSprint Database* [39]	The database provides information about hotspots in protein interface using conservation rate and solvent accessibility of the residues.	Numb phosphotyrosine-binding domain [39]	http://prism.ccbb.ku.edu.tr/hotsprint/
	*HotSpot wizard* [41]	The web server predicts residue mutability of functionally important residues and visualizes it on protein sequence and structure.	Haloalkane dehalogenase, Phosphotriesterase, 1,3-1,4-b-D-Glucan 4-glucanohydrolase, β-Lactamase [41]	http://loschmidt.chemi.muni.cz/hotspotwizard/
	*Selecton* [42]	The web server detects selection forces on biologically significant sites in the target protein during evolutionary process.	TRIM5α protein [42]	http://selecton.tau.ac.il/index.html

Protein superfamily based MSA	*3DM* [37]	The database performs structure based MSA for a protein superfamily with sequence, structural, molecular interaction and mutational information from the literature.	α/β hydrolase fold [53]	http://3dmcsis.systemsbiology.nl/
	*The Lipase Engineering Database* [43, 54, 55]	The database performs protein superfamily based MSA and annotates functionally relevant amino acid positions with structural and mutational information.	Lipases [43, 54, 55]	http://www.led.uni-stuttgart.de/
	*The database of epoxide hydrolases and haloalkane dehalogenase* [56]		Epoxide hydrolases and haloalkane dehalogenase [56]	http://www.led.uni-stuttgart.de/
	*The Laccase Engineering database* [45]		Laccases [45]	http://www.lcced.uni-stuttgart.de/
	*The Cytochrome P450 engineering database* [57]		Cytochrome P450s [57]	http://www.cyped.uni-stuttgart.de/
	*The PHA Depolymerase Engineering Database* [44]		Polyhydroxyalkanoates depolymerase [44]	http://www.ded.uni-stuttgart.de/
	*The Lactamase Engineering database* [46]		Lactamases [46]	http://www.laced.uni-stuttgart.de/
	*SHV Lactamase Engineering Database* [47]		SHV lactamases [47]	http://www.laced.uni-stuttgart.de/classA/SHVED/

Literature based protein mutant data	*PMD* [48]	The database provides literature based protein mutant information with structure and functional annotation.		http://pmd.ddbj.nig.ac.jp/∼pmd/pmd.html
	*ProTherm* [49–51]	The database provides literature based protein mutant information with thermodynamic parameters and experimental conditions integrated with sequence, structure and function annotation.		http://gibk26.bio.kyutech.ac.jp/jouhou/Protherm/protherm.html
	*MuteinDB* [52]	The database provides literature based protein mutant information, kinetic parameters and experimental conditions integrated with user-friendly and flexible query system to fetch data using reaction name or substrate or inhibitor name or structure and mutations.	Cytochrome P450s [52]	https://muteindb.genome.tugraz.at/muteindb-web-2.0/faces/init/index.seam

For more focused analysis of protein hotspots or amino acid patches, three interesting tools are available as standalone programs or web servers. The *Joint Evolutionary Tree* (*JET*) method is more tuned to identify the conserved amino acids patches on protein interface by taking into account the physical-chemical properties and evolutionary conservation of the surface residues [38]. The predicted protein interaction sites or core residues might be used in site-specific mutagenesis experiments. *HotSprint* [39] database provides information of the hotspots in protein interfaces using the sequence conservation score (calculated by Rate4Site algorithm [40]) of the residues and their solvent accessible surface area. *HotSpot Wizard* predicts the suitability of the mutagenesis of the amino acids in or near the active site using their evolutionary conservation information [41]. The server takes protein structure as input and provides a platform to experimentalists to select target amino acids for site directed mutagenesis to improve enzymatic properties like substrate specificities, activity and enantioselectivity [41]. *MAP*^*2.0*^*3D* [27] (Table 1, see previous paragraph) also provides the information of mutagenic hotspots generated due to the mutational preferences of the random mutagenesis methods with sequence and structural information of protein. *Selecton* [42] web server predicts the selective forces at each amino acid position in protein. The server performs the codon-based alignment on a set of the homologous nucleotide sequences and uses the ratio of amino acids altered to silent substitutions (Ka/Ks) to estimate both the positive (>1) and purifying (<1) selections. These values are then projected on the primary sequence or, if available, on the tertiary structure of the protein to detect variability in biologically significant sites. These sites may be interpreted as being the consequences of molecular adaptations, which confers an evolutionary advantage to the organism.

A series of protein superfamily specific databases (see Table 2), containing selected enzymes relevant to protein engineering applications, have been introduced by Pleiss *et al*. Functionally relevant residues are annotated followed by MSA of protein sequences or structures of the superfamily with published protein mutation data to derive sequence-structure-function relationships [43–47]. *PMD* [48] (Protein Mutation Database), *ProTherm* [49–51] and *MuteinDB* [52] are literature based databases of protein and its mutant information that are integrated with sequence and structure information. *ProTherm* [49–51] database also includes experimental thermodynamic and kinetic parameters (e.g. Gibbs free energy changes of unfolding, heat capacity changes, and protein activities) of wild-type and their mutants. *MuteinDB* [52] stores and provides enzyme mutant data with their catalyzed reaction, kinetics (K_m_ and K_cat_) and experimental conditions. The database has a user-friendly and flexible query system to use reaction, substrate, mutation or inhibitor to fetch the information.

## Structure-based focused library

The structure based approaches assist rational design and random mutagenesis by predicting regions in the protein responsible for stability and activity [2, 58]. The computational tools as *3DLigandSite* [59], *ProBiS* [60, 61] (Protein Binding Site) and *SiteComp* [62] predict ligand binding site in protein [63]. All these tools, in the absence of crystal structure, use the homology model of the target protein and aid the design and tune ligand binding site by identifying key residues for activity and their molecular interactions properties. *3DLigandSite* [59] performs alignment and clustering of the homologous structures to predict ligand binding site. *ProBiS* [60, 61] uses MSA to detect structurally similar binding site in protein and also perform local structural pairwise alignment to identify functionally relevant binding regions. The pre-calculated results of *ProBiS* analysis are available via *ProBiS-database* [64] as a repository of structurally similar binding sites. *SiteComp* [62] characterizes protein binding site using molecular interaction fields based descriptors. The server evaluates differences in similar binding sites, identification of sub-sites and residue contributions in ligand binding. *TRITON* [65, 66] provides the single platform to protein engineers to model mutants, perform protein-ligand docking and calculate reaction pathways. In this way, these methods facilitate to study the properties of protein-ligand complexes.

The knowledge of molecular interactions, contribute to relevant free energy barrier, and the design of surface charge distribution, can help to understand the molecular basis of kinetic stability and efficiently modulates the enhancement of protein stability [58, 67]. *PIC* (Protein Interaction Calculator) server [68] calculates inter or intra protein interactions using published criteria integrated with solvent accessibility and residue depth calculations. The web server, *COCOMAP* (bioCOmplexes COntact MAPs) [69] uses intermolecular interactions to analyze interfaces in biological complexes. The identification of exposed and buried amino acids also helps to gain insight into protein stability and to explore the mutational effect on protein. *DEPTH* [70] employ distance information between residues and bulk solvent to predict protein stability, conservation or binding cavity based on information about residue depth and solvent accessibility. *SRide* [71] provides residual contribution to protein stability using interactions, evolutionary conservations and hydrophobicity of their neighboring residues. *Patch finder plus* [72] identifies residues that contribute to positively charge patches on protein surface and might interact with DNA, membrane or the other protein. *ConPlex* [73] utilizes protein solvent accessible surface area to identify surface or interface residues and assign residue specific conservation score on sequence and structure of the protein complex. The server also provides the pre-calculated *ConPlex* results of known protein complexes as repository.

Recent studies have suggested that protein flexibility and protein functions are strongly linked [24, 74, 75]. Protein flexibility plays an important role in both catalytic activity and molecular recognition processes. The effect of protein flexibility is particularly relevant in protein from extremophiles to balance rigidity required for stability and flexibility necessary for activity [76–78]. In addition, numerous proteins have regions, adopt different conformation under different conditions, allowing them to take part in cellular and molecular regulation [24, 79]. The residue flexibility in protein has been taken in account to describe a variety of protein properties including relation with thermal stability, catalytic activity, ligand binding (induced fit), domain motion, preferential solvation and molecular recognition in intrinsically disordered protein system. The Debye–Waller factor, reported in crystallographic atomic resolution structures, provides a rough estimation of local residue flexibility [80] and different servers provide this information as an indicator (for example, in *MAP*^*2.0*^*3D* server [27]). If the crystallographic structure is not available then different tools can be used to estimate flexibility profiles using different approaches.

The *RosettaBackrub* [81] server can generate protein backbone structural variability as consequence of amino acid variations [82] that can be used to design sequence libraries for experimental screening and to predict protein or peptide interaction specificity. The server generates Rosetta scored modeled structures for variant with single or multiple point mutations in monomeric proteins. It also generates near-native structural ensembles of protein backbone conformations and sequences consistent with those ensembles. Finally, it can predict sequences tolerated by proteins or protein interfaces using flexible backbone design methods. The *tCONCOORD* [83] method generates conformational ensembles to gain insight in the conformational flexibility and conformational space of the protein.

*FlexPred* [84] specially predicts residue flexibility using pattern recognition approach to identify residue positions in conformations switches integrated with their evolutionary conservation and normalized solvent accessibility (if structure is available) as the Support Vector Machine (SVM) predictors.

Different simplified methods have been proposed to identify local flexibility or large scale motions in protein at coarse-grained level [85–87] Many of these methods are based on Gaussian network model (GNM) [88] or its extension, the anisotropic network model (ANM) [89] to study protein dynamics using Normal Mode Analysis (NMA) (see the review [90] for a general overview about these topics). Table 3 shows the tools available to analyze conformational flexibility on protein structure (for more details see [91]). *ElNemo* [92] and *WEBnb@* [93] servers are reported here to complete the information about NMA based tools. Both the servers perform NMA using coarse grain model to analyze the conformational changes in protein. *FlexServ* [94] server estimates protein flexibility using three different coarse-grained approaches: 1) discrete molecular dynamics (DMD), 2) normal mode analysis (NMA) and 3) Brownian dynamics (BD). The server characterizes protein flexibility by analyzing different structural and dynamic properties of the protein such as structural variations, essential modes, stiffness between the interacting residues and dynamic domains and hinge points. Different tools are available to identify hinge bending residues on large-scale protein motions. *HINGEprot* [95] server predicts hinge motion in protein using coarse grained GNM and ANM model. *DynDom* [96] use a rigorous approach to describe domain motion. The method determines hinge axes and hinge bending residues using two conformations of the protein. A recent addition to DynDom is the ligand-induced domain movements in enzymes database^97^. Furthermore, the *Dyndom3D* [98] server provides a more advanced and generic tool that can be used to study any kind of polymer.

**Table 3:** Summarizing the computational tools for structure-based focused library generation

Approach	Name	Description	Case study examples	URL
Ligand binding site	*3DLigandSite* [59]	The web server identifies ligand binding site via MSA and clustering algorithm.	Target T0483 in CASP8	http://www.sbg.bio.ic.ac.uk/∼3dligandsite/
	*ProBiS* [60, 61]	The web server detects binding site using MSA and characterizes it using local structural pairwise alignment.	Biotin carboxylase, TATA binding protein [60], D-alanine–D-alanine ligase, Protein kinases C [61]	http://probis.cmm.ki.si/
	*ProBiS-database* [64]	The database provides structurally similar protein binding site using *ProBiS* algorithm.	Cytochrome c [64]	http://probis.cmm.ki.si/?what=database
	*SiteComp* [62]	The web server characterizes ligand binding site using molecular interaction descriptors.	Cyclooxygenase, adenylate kinase [62]	http://scbx.mssm.edu/sitecomp/sitecomp-web/Input.html
	*TRITON* [65, 66]	The method facilitates to model mutant, dock ligand in the protein and calculates reaction pathways for the characterization of protein-ligand interactions using Semi-empirical quantum-mechanics approach.	PA-IIL lectin and its mutants [65]	http://www.ncbr.muni.cz/triton/description.html

Protein interaction	*PIC* [68]	The web server calculates the molecular interactions using published criteria.	-	http://pic.mbu.iisc.ernet.in/job.html
	*COCOMAPS* [69]	The web server analyzes and visualizes interfaces in biological complexes using intermolecular contact maps based on distance or physicochemical properties.	Hen egg lysozyme interaction with two antibodies [69]	https://www.molnac.unisa.it/BioTools/cocomaps/

Residue depth and stability	*DEPTH* [70]	The web server predicts binding cavity and mutational effect on protein stability using residue depth and solvent accessible surface area.	West Nile Virus NS2B/NS3 protease [70]	http://mspc.bii.a-star.edu.sg/tankp/intro.html
	*SRIde* [71]	The web server predicts the contribution of residues in protein stability using interactions with its spatial neighbors and their evolutionary conservation.	TIM-barrel proteins [103]	http://sride.enzim.hu/

Protein surface and interface	*Patch finder plus* [72]	The web server identifies large positively charged electrostatic patches on protein surface using Poisson Boltzmann electrostatic potential.	DNA binding domain of TATA binding protein [72]	http://pfp.technion.ac.il/
	*ConPlex* [73]	The web server performs evolutionary conservation analysis of the protein complex.	Rho–RhoGAP complex [73]	http://sbi.postech.ac.kr/ConPlex/

Protein flexibility	*RosettaBackrub* [81]	The web server performs flexible backbone modeling using Backrub [104] method to design tolerated protein sequences.	hGH-hGHr interface [105]	https://kortemmelab.ucsf.edu/backrub/cgi-bin/rosettaweb.py?query=index
	*tCONCOORD* [83]	The method generates conformation ensemble and transitions using geometrical constrains based prediction of protein conformational flexibility.	Osmoprotection protein [83]	http://wwwuser.gwdg.de/~dseelig/tconcoord.html
	*FlexPred* [84]	The web server predicts residue flexibility in the protein using SVM approach.	Human PrP [106]	http://flexpred.rit.albany.edu/
	*ElNemo* [92]	The web server predicts large amplitude motions in the protein using NMA.	HIV-1 protease, *E. coli* membrane channel protein TolC	http://igs-server.cnrs-mrs.fr/elnemo/index.html
	*WEBnm@* [93]		Calcium ATPase [93]	http://apps.cbu.uib.no/webnma/home
	*FlexServ* [94]	The web server determines and analyzes protein flexibility using coarse-grained modeling approach.	-	http://mmb.pcb.ub.es/FlexServ/input.php
	*HINGEprot* [95]	The web server detects hinge region in the protein using both GNM and ANM.	Calmodulin protein, hemoglobin [95]	http://www.prc.boun.edu.tr/appserv/prc/hingeprot/
	*DynDom3D* [98]	The web server predicts domain motions using conformational changes in the protein.	Hemoglobin, 70S ribosome [98]	http://fizz.cmp.uea.ac.uk/dyndom/3D/

The reader should be noticed that the connection between protein flexibility and function has been investigated theoretically and experimentally only in the last few years [87, 99–101]. The methods based on this approach provide a qualitative estimation of protein dynamical properties but they do not take into account many effects (such as direct solvent effects) that are important for protein functionality. Till now, the atomistic simulation (MD or QM/MD) is the best approach to quantitatively study protein flexibility and dynamics [8, 99, 102]. Nevertheless, even to this level of accuracy, the connection between flexibility and functionality is still puzzling. In addition, the simulation approaches are still time consuming and unpractical for high-throughput modeling and analysis of protein structural dynamics.

## Mutational effects in protein

For biotechnological applications, the enhancement of protein thermal stability or tolerance is a common requested task in protein engineering [107]. Highly stable structure correlates with well-packed highly compact structure and has increased tolerance to mutation because mostly the mutations are deleterious i.e. related to instability of protein [108]. Generally the effect of the mutation on protein has been calculated by the free energy differences between two states of protein like thermodynamic stability as change in free energy in folded and unfolded state (▵▵G). The mutational effect has been predicted by using different machine learning and selection methods (as SVM, Decision Tree (DT) or Random Forest (RE) [109]) for classification or regression of data or by using statistical or empirical methods taking into account the atomic interactions or structural properties like solvent accessibility. Most of the servers based on these approaches use available information of mutational effects (fetched from databases like *PMD* [48], *ProTherm* [51]) to predict the effect of new substitutions. Table 4 summarizes the available tools to predict mutational effects on protein stability and activity using different methods. *I-Mutant2.0* [110] and *MUpro* [111] are SVM based methods to predict stabilizing or destabilizing amino acid substitutions based on free energy change (▵▵G). *iPTREE-STAB* [112] server employ a DT approach to predict the effect of single point mutations on protein stability considering physicochemical properties and contact information of the substituted amino acid with their neighboring amino acids. *WET-STAB* [113] server performs a similar prediction with an additional feature to predict protein stability changes upon double mutations from amino acid sequence. *ProMAYA* [114] uses RF machine learning algorithm to predict protein stability based on free energy difference. *MuD* (Mutation detector) uses the same algorithm for the classification of amino acid substitutions as neutral or deleterious by taking into account structure- and sequence-based features as solvent accessibility, binding site, sequence identity [115]. *SDM* (Site Directed Mutator) [116] and *PopMuSic2.1* [117] are statistical derived force field potential based methods for protein stability prediction using relative free energy differences. In *PopMuSic2.1* [117], however, the parameters of statistical derived force field potential depend on protein solvent accessibility. *FoldX* plugin [118] and *PEAT-SA* [119] program suite utilize empirical force field to calculate, from three-dimensional protein or peptides structures, the relative free energy difference determined by the changes of interactions in the mutated structures. *CUPSAT* [120] estimates the effect of mutations on the protein stability using protein environment specific mean force potentials. The potentials are derived from statistical analysis of protein structure data sets. *AUTO-MUTE* [121, 122] provides either energy based or machine learning methods for the prediction of protein stability by providing protein structure, mutation and experimental condition. *SIFT* (Sorts Intolerant From Tolerant) [123] server helps to explore the effect of mutation on protein function using sequence homology approach. The multiple alignment information is used to identify tolerated and deleterious substitutions in the query sequence.

**Table 4:** Summarizing the computational tools to analyze the mutational effect on protein stability and activity.

Approach	Name	Description	URL
SVM	*I-Mutant2.0* [110]	The web server predicts protein stability change upon point mutation.	http://folding.uib.es/i-mutant/i-mutant2.0.html
	*MUpro* [111]		http://mupro.proteomics.ics.uci.edu/
Decision tree (DT)	*iPTREE-STAB* [112]	The web server predicts protein stability change with residues information.	http://210.60.98.19/IPTREEr/iptree.htm
	*WET-STAB* [113]	The web server predicts protein stability change upon double mutation with residue information.	http://210.60.98.19/WETr/wet.htm
Random forests (RF)	*ProMAYA* [114]	The web server predicts mutational effect on protein function.	http://bental.tau.ac.il/ProMaya/
	*MuD* [115]		http://mud.tau.ac.il/
Statistical potential based method	*SDM* [116]	The web server predicts mutational effect on protein stability.	http://mordred.bioc.cam.ac.uk/sdm/sdm.php
	*PopMuSic2.1* [117]	The web server predicts thermodynamic stability change upon mutation.	http://babylone.ulb.ac.be/popmusic/
Empirical force field	*FoldX* [118]	The plugin predicts mutational effect on protein and facilitates *in- silico* alanine screening, mutant homology modeling and interaction energy calculation.	http://foldx.crg.es/
	*PEAT-SA* [119]	The program suite predict mutational effect on protein stability, ligand affinity and pKa values.	http://enzyme.ucd.ie/PEATSA/Pages/FrontPage.php
	*CUPSAT* [120]	The web server predicts mutational effect on protein stability.	http://cupsat.tu-bs.de/
RF, SVM, Tree and SVM regression	*AUTO-MUTE* [122]	The web server predicts mutational effect on protein stability and activity (up to 19 mutations).	http://proteins.gmu.edu/automute/
Evolutionary conservation	*SIFT* [123]	The web server predicts mutational effect on protein function.	http://sift.jcvi.org/

A quantitative *in-silico* screening of the virtual libraries based on the cooperative effect of multiple mutations to the stability and functionality is still out of reach. However, the current methods allow a qualitative indication of possible mutation sites that can increase the chances to get higher population of stable and functionally active variants in the library. The available knowledge of mutational effects on protein provided by all these CAPDE approaches help to limit library size and focus to generate unpredictable substitutions that may lead to large effects. These libraries based on *in-silico* screening generally show a higher success rate when the starting protein has sufficient stability.

## Summary and Outlook

In this review, the recent additions to the CAPDE arsenal of computational tools, servers and databases have been briefly reviewed. The rapid accumulation of the knowledge on protein structures and sequence-structure-function relationships foresees the continuous amelioration of these methods. In particular, machine-learning approaches, in which the volume of data is the heuristic key to access the hidden knowledge, statistical based force fields for coarse-grained approaches will surely benefit this trend. These approaches are not only the convenient aids to support lab experiments but also the workbench for heuristically blueprinting novel molecules. In addition, the availability of the low cost and high performance computers will soon transform currently expensive physically based simulations to the convenient and very accurate high throughput computational tools. This will make possible to predict structural stability and folds of small or medium sized proteins and will open a new working style paradigm in protein engineering. In addition, physical based approaches have recently shown promising results to understand enzyme activity [124, 125].
